# A parathyroid carcinoma within a cold thyroid nodule

**DOI:** 10.3332/ecancer.2009.150

**Published:** 2009-09-18

**Authors:** LL Travaini, G Trifiro, G Paganelli

**Affiliations:** Nuclear Medicine Division, European Institute of Oncology, Via Ripamonti 435, 20141 Milan, Italy

## Abstract

We report the case of a 71-year-old woman who was referred to our institute with a solid nodule in the right thyroid lobe and hypercalcemia. Ultrasound revealed a well-vascularized right thyroid nodule that was identified as a cold area by ^99m^Tc-sodium pertechnetate scan. Fine-needle aspiration showed a follicular lesion and blood tests revealed hypercalcemia and hyperparathyroidism. A ^99m^Tc-methoxyisobutylisonitrile (^99m^Tc-Sestamibi) scan was subsequently performed revealing a focal area of increased uptake in the right thyroid lobe, within the cold area detected by the thyroid scan. A right emithyroidectomy and right superior and inferior parathyroidectomy was performed and histopathological examination showed a parathyroid carcinoma (immunohistochemistry positive for PTH and chromogranin A, Ki-67 10%) associated with follicular hyperplasia.

A 71-year-old woman was referred to our institute with a solid nodule in the right thyroid lobe combined with hyperparathyroidism and hypercalcemia. She had a prior history of infiltrating ductal carcinoma (pT1c pN0) for which she had a right quadrantectomy and axillary lymph node dissection.

Ultrasound (US) revealed a 3.5 × 2.7 × 2.5 cm well-vascularized nodule in the right thyroid lobe, characterized by cystic and solid components. Fine-needle aspiration indicated a follicular lesion.

A ^99m^Tc-sodium pertechnetate scan showed a large cold area in the right thyroid lobe (Figure 1A).

Blood examination revealed a calcium level of 14.5 mg/dl (normal range 8.1–10.4), a phosphate level of 2.1 mg/dl (normal range 2.7–4.5), a parathyroid hormone (PTH) level of 4480 pg/ml (normal range 14.0–72.0) and normal levels of TSH and thyroglobulin.

A ^99m^Tc-methoxyisobutylisonitrile (^99m^Tc-Sestamibi) scan subsequently performed revealed a focal area of increased uptake in the right thyroid lobe, within the cold area detected by the thyroid scan (Figure 1B and C) and is clearly evident in the subtraction image (Figure 1D). This area of increased uptake indicated tissue with high-mitochondrial activity probably of parathyroid origin.

A right hemithyroidectomy and right superior and inferior parathyroidectomy was performed. Histopathological examination showed a parathyroid carcinoma (immunohistochemically positive for PTH and chromogranin A, Ki-67 10%) associated with follicular hyperplasia.

## Discussion

Primary hyperparathyroidism (hPTH) is a disorder that is usually treated by surgery.

In the past, the standard was bilateral neck exploration, but, recently, there has been rapidly developing interest in less invasive surgical management because of the evolution of certain imaging techniques [1–5], which allow precise, preoperative localization of the lesion. ^99m^Tc-Sestamibi, which was initially introduced for cardiac scintigraphy, was accidentally found to accumulate in parathyroid adenomas [6]. The exact mechanism of its elective uptake in abnormal parathyroid glands remains debatable. High-mitochondrial activity is considered to be the major component of Sestamibi uptake by human parathyroid tissue in patients with pHPT [7,8].

This functional imaging has very high specificity for parathyroid tissue, with only a small number of reports of false-positive uptake into lymph nodes or thyroid nodules [9,10].

High-frequency ultrasonography imaging is a highly sensitive technique [11] in experienced hands, but in patients with thyroid nodules, it is less precise [12–14] and is often unable to differentiate between benign and malignant thyroid nodules.

Although there are only a few reports [15,16], which have addressed the diagnostic importance of US for thyroid disease associated with an increase of hPTH, Mihai *et al* [17] suggest that in cases of hPTH the combined use of ^99m^Tc-Sestamibi and US are two complementary investigating tools.

However, no clear consensus has been reached regarding which protocol to use in such cases.

Reports of concomitant thyroid disease in patients with hPTH date to the early 1950s, with a prevalence ranging from 22% to 70% [16,18,19]. The cause of this association remains controversial: some authors described it as incidental [20], meanwhile others suggest the presence of goitrogenic factors, such as increased endogenous calcium, epithelial growth factor or moreover insulin-like growth factor [19,21,22], may influence the development of thyroid disease. Recently, Masatsugu *et al* [23] reported that thyroid disease (including non-medullary thyroid carcinoma) occurs frequently in the presence of sporadic hPTH and underlined the importance of pre-operative US for evaluating possible concomitant thyroid disease, especially malignant disease, in order to determine appropriate surgical management.

Here, we report the clinical history of a patient who was suspected to have a follicular thyroid carcinoma (cytology and US findings) with concomitant (incidental) hPTH. A ^99m^Tc-Sestamibi scan identified an area of increased mitochondrial activity (suggesting parathyroid involvement), overlying an area already reported by US, in the right thyroid lobe.

Unequivocally, the surgery should have been the final decision because of hPTH but, in this particular patient, the US and ^99m^Tc-Sestamibi scans were discordant on the tissue origin and the cytological diagnosis was wrong.

## Conclusion

Imaging in patients with primary hyperparathyroidism has been proven difficult. We suggest that the combination of ^99m^Tc-Sestamibi scintigraphy, and US may be more informative in such cases.

US, although operator dependent, is relatively cheap and should be carried out first in order to examine the thyroid morphology, the ***in situ*** parathyroid glands, when visible, and as a guide for the cytologist. A ^99m^Tc-Sestamibi scan is necessary to detect *extra-situ* parathyroid glands and to characterize thyroid and parathyroid nodules.

## Figures and Tables

**Figure 1:**
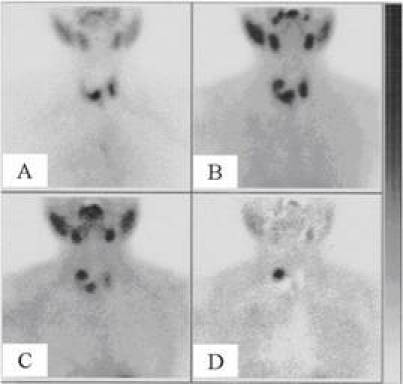
(A) [^99m^Tc] sodium pertechnetate scan shows a cold area in the upper right thyroid lobe. Early (B) and delayed (C) [^99m^Tc] Sestamibi scans reveal a focal area of increased uptake in the upper right thyroid lobe, within the cold area detected by the thyroid [^99m^Tc] sodium pertechnetate scan. (D) Processed image obtained by data subtraction of the [^99m^Tc] Sestamibi scan from the [^99m^Tc] sodium pertechnetate scan.
